# Unintended Pregnancies in Brazil - A Challenge for the Recommendation to Delay Pregnancy Due to Zika

**DOI:** 10.1371/currents.outbreaks.7038a6813f734c1db547240c2a0ba291

**Published:** 2016-03-16

**Authors:** Cynthia Schuck-Paim, Daniel López, Lone Simonsen, Wladimir Alonso

**Affiliations:** Wolfson College, Oxford University, Oxford, Oxfordshire, United Kingdom; National Institute of Pure and Applied Mathematics (IMPA), Rio de Janeiro, Brazil; Department of Global Health, George Washington University, Milken Insittute School of Public Health, Washington DC, Maryland, USA; Laboratory for Human Evolution Studies, Genetics and Evolutionary Biology, University of São Paulo, São Paulo, Brazil

**Keywords:** Aedes, Brazil, microcephaly, pregancy, teenagers, Zika

## Abstract

Because of the potential link between the ongoing Zika virus outbreak and a surge in the number of cases of congenital microcephaly, officials in Latin America have recommended that women postpone pregnancy until this association is firmly established or the outbreak subsides. However, in all these countries a large proportion of babies are still born out of unplanned pregnancies. Teenage girls are particularly at high risk, as they often lack access to preventive contraception methods, or the knowledge to use them appropriately. To gauge the magnitude of the barriers preventing the implementation of such a recommendation in Brazil, the country so far most affected by the Zika epidemic, we evaluated pregnancy rates in teenage girls, and their spatial heterogeneity in the country, in recent years (2012-2014). Nearly 20% of children born in Brazil today (~560,000 live births) are by teenage mothers. Birth incidence is far higher in the tropical and poorer northern states. However, in absolute terms most births occur in the populous southeastern states, matching to a large extent the geographic distribution of dengue (an indicator of suitable climatic and sociodemographic conditions for the circulation of Aedes mosquitoes). These findings indicate that recommendation to delay pregnancy will leave over half a million pregnant adolescents in Brazil vulnerable to infection every year if not accompanied by effective education and real access to prevention.

## Unintended pregnancies in Brazil - A challenge for the recommendation to delay pregnancy due to Zika

The World Health Organization has recently declared the emerging Zika virus an international public health emergency^1^. The virus, which reached Brazil to cause a major epidemic starting in April 2015, has rapidly spread to over 28 countries^2^ and may still reach many more considering the widespread distribution of its vector species (*Aedes aegypti* mosquitoes) and the high transmission rates observed so far. Concerns also involve the possibility of a second vector of Zika transmission, *Aedes albopictus*, which is currently expanding its ecological niche globally 3 ^,^ 4 .

However, the biggest concern about the Zika outbreak is the potential link with the surge of reported cases of microcephaly and other malformations of the central nervous system in newborns from mothers infected with the virus during pregnancy^1^. The proposed link between Zika infection and congenital microcephaly is not conclusive^1^^,^ 5, but given the long-term consequences of this birth defect (which can range from mild developmental delays to severe, lifelong motor and cognitive impairment), a number of Officials in Latin America have recommended that women avoid or defer pregnancy, giving time for the epidemic to subside and therapies to develop^6^. Among these were representatives of the Health Ministries of Ecuador, Colombia, Jamaica and El Salvador. In Brazil the recommendation to postpone pregnancy has not been made official, yet the director of the Surveillance Department at the Ministry of Health - Cláudio Maierovitch - has stated that women from high-risk areas who can wait to conceive should do so^2^.

The implementation of such a recommendation requires, however, that women in these areas are not only clearly informed about the risks posed by infection, but also have control over their reproductive choices. There are few reasons to doubt that most women will be aware and informed about the disease and its consequences through the widespread media. Yet ensuring control over the timing of pregnancy is a more daunting challenge, especially considering that many babies are born out of unplanned pregnancies - even when strong motives for avoiding them exist (e.g. age, economic, social, professional and health-related).

In Brazil, the country most affected by the Zika outbreak so far, data from a demographic survey from 2006 indicate that approximately half of all births that happened five years prior to the survey were unplanned^7^. A demographic group at particularly high risk is that of adolescents (defined as the period from ten to 19 years old, following the World Health Organization definition^8^). Pregnancies in adolescent girls are far more likely to be unplanned because this group often lacks access to contraception and family planning methods, or the knowledge to use them appropriately. In such circumstances, the risk of conceiving a child with congenital anomalies might not affect the likelihood of a pregnancy.

To gauge the magnitude of the barriers preventing the postponement of parenthood in Brazil, we evaluated pregnancy rates in adolescent groups, and their spatial heterogeneity in the country, in recent years for which data are available (2012-2014). Data on live births were obtained from the Information System on Live Births (SINASC^9^). Population estimates for each state and age group were obtained from the Brazilian Institute of Geography and Statistics^10^ and annual population data were calculated by spline interpolation of census data. Analyses were conducted using the freely available analytical software Epipoi^11^.

Although Brazil has experienced a substantial decline in fertility rate and a corresponding displacement of reproduction towards older ages over the last decades, nearly 20% of children born in Brazil today are by teenage mothers, a proportion nearly twice as high as the world average (11%) in this age group^12^. Every year, over 560,000 children are born to adolescent mothers in Brazil (Fig.1).

**Mean number of live births per year (2012-2014) from adolescent mothers in each Brazilian state (source: Datasus: SINASC) and number of dengue cases in 2015 (source: SVS, Brazlian Ministry of Health).**
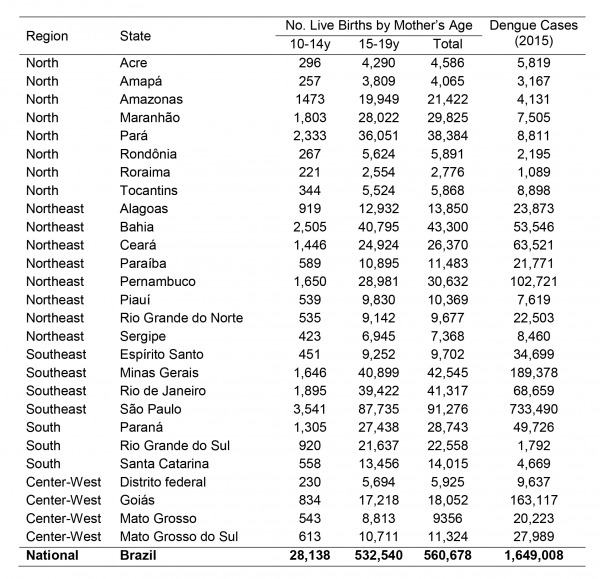

The states with the highest incidence of births from adolescents in Brazil are those in the northern region (coinciding with a large part of the Amazon area) and Mato Grosso do Sul (Fig.2). The pattern is similar for mothers aged 10-14 years (Fig.2a) and 15-19 years (Fig.2b). When birth incidence among adolescent mothers (10-19y) is plotted against latitude (Fig.3), it is clear that it increases with decreasing latitudes, being far higher in the tropical northern states. In absolute terms, however, most births occur in the most populous southeastern states, most notably São Paulo (Fig.1). The distribution of dengue cases in 2015 by state is shown in Figure 1, as an indicator of those states with climatological and sociodemographic suitable conditions for the circulation of Aedes mosquitoes. As shown in Fig.1, the highest number of dengue cases also occur in populous states in the southeast (predominantly São Paulo), matching to a large extent the spatial distribution of the absolute number of births from adolescent mothers.

**Monthly incidence of live births (per 100,000 age-specific population) from mothers aged (A) 10-14 years and (B) 15-19 years in each Brazilian state.**
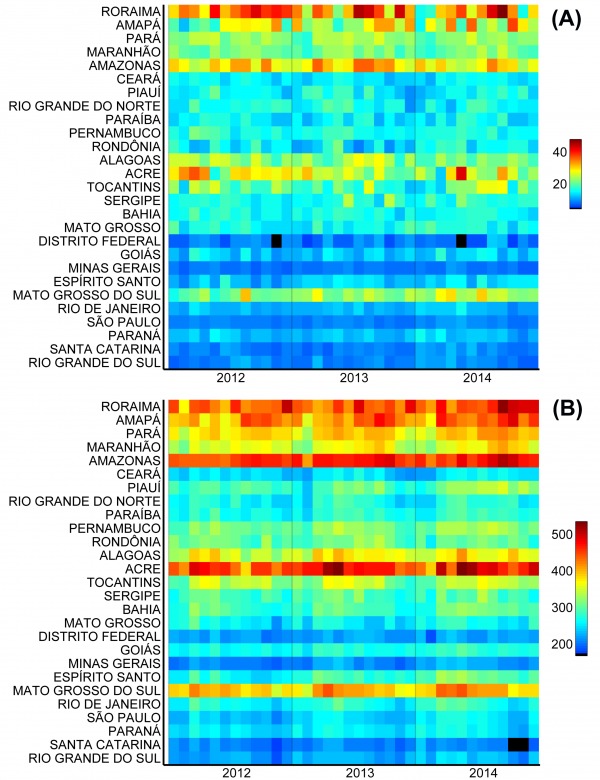

**Average incidence of live births (per 100,000 age-specific population; 2012-2014) from mothers aged 10-19 years in each Brazilian state by the latitude of the state’s capital:**
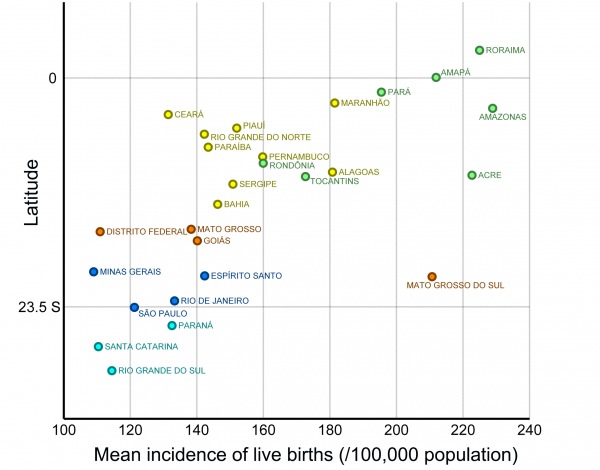

The pre-existing high incidence of what are most likely unplanned births by adolescent mothers in high-risk areas emphasizes that the recommendation to delay pregnancy during the current crisis is only a partial solution to protect babies from the putative effects of infection. Unless accompanied by changes in the accessibility to contraception methods and a drastic change in health education, access to and attitudes towards family planning, the recommendation to delay pregnancy will leave over half a million pregnant adolescents vulnerable every year until effective interventions (including vaccines) to prevent Zika infection are not available^13^.

More complicated yet is the communication of risk at a time when microcephaly has not been firmly linked to Zika infection. While we await the final verdict of the magnitude of risk and strength of association, recommendations related to conception in the time of Zika should be approached with care, health education and real access to prevention.

## Competing Interest

The authors have declared that no competing interests exist.
